# Genome editing in macroalgae: advances and challenges

**DOI:** 10.3389/fgeed.2024.1380682

**Published:** 2024-03-06

**Authors:** Jonas De Saeger, Emma Coulembier Vandelannoote, Hojun Lee, Jihae Park, Jonas Blomme

**Affiliations:** ^1^ Bio Environmental Science and Technology (BEST) Lab, Ghent University Global Campus, Yeonsu-gu, Republic of Korea; ^2^ Department of Biology, Phycology Research Group, Ghent University, Ghent, Belgium; ^3^ Department of Plant Biotechnology and Bioinformatics, Ghent University, Ghent, Belgium; ^4^ VIB-UGent Center for Plant Systems Biology, Ghent, Belgium

**Keywords:** genome editing, CRISPR, macroalgae, seaweed biotechnology, seaweed breeding, reverse genetics

## Abstract

This minireview examines the current state and challenges of genome editing in macroalgae. Despite the ecological and economic significance of this group of organisms, genome editing has seen limited applications. While CRISPR functionality has been established in two brown (*Ectocarpus* species 7 and *Saccharina japonica*) and one green seaweed (*Ulva prolifera*), these studies are limited to proof-of-concept demonstrations. All studies also (co)-targeted *ADENINE PHOSPHORIBOSYL TRANSFERASE* to enrich for mutants, due to the relatively low editing efficiencies. To advance the field, there should be a focus on advancing auxiliary technologies, particularly stable transformation, so that novel editing reagents can be screened for their efficiency. More work is also needed on understanding DNA repair in these organisms, as this is tightly linked with the editing outcomes. Developing efficient genome editing tools for macroalgae will unlock the ability to characterize their genes, which is largely uncharted terrain. Moreover, given their economic importance, genome editing will also impact breeding campaigns to develop strains that have better yields, produce more commercially valuable compounds, and show improved resilience to the impacts of global change.

## 1 Introduction

Multicellular marine macroalgae are typically classified into the red (Rhodophyta), brown (Phaeophyta) and green (Chlorophyta) seaweeds (Littler and Littler, 2011; Pereira, 2021). Their shared designation as seaweeds, however, belies the profound evolutionary divergence among these groups, as the lineages of photosynthetic organisms have split at the root of the eukaryotic tree ∼2 billion years ago (Strassert et al., 2021). The ecological and economic significance of these organisms cannot be overstated. As primary producers, seaweeds play a pivotal role in marine ecosystems. Furthermore, wild harvested or cultured individuals produce food, feed, fuel, and useful chemicals. This versatility underpins a robust seaweed farming industry, which boasts an estimated global value of 14.7 billion USD (Cai et al., 2021). On the other hand, certain seaweed genera are noted for their less favorable effects, including biofouling and massive blooming (Coates et al., 2015).

Despite their importance, seaweed research has only recently entered the era of genomics and molecular biology. Since the first seaweed nuclear genome of *Ectocarpus* species 7 was reported (Cock et al., 2010), many representatives of different seaweed groups have been sequenced (reviewed in Stock et al. (2024); Table 1). Nevertheless, these genomes remain a black box. When Blaby-Haas and Merchant (2019) assessed about 100 genomes of micro- and macroalgae, they found that over 50% of the genes were of unknown function. Although genome data is crucial for understanding seaweed biology, functional characterization of genes in these diverse species remains largely unexplored. To bridge this gap, one approach to perform functional analysis of genes is studying mutants that occur either naturally or by induction (Table 1). In green algae, mutations have been found that affect cell division, vegetative development, or result in sterility (Bryhni, 1974; Kakinuma et al., 2006; Jongma et al., 2013; Oertel et al., 2015; Gao et al., 2017). Fast growing, differently pigmented, or high monospore-producing mutants were isolated from a number of different red algae genera (Kursar et al., 1983; Patwary and Meer, 1983; Niwa et al., 1993; Yan et al., 2000; Plastino et al., 2004; Cornish et al., 2013; Lee and Choi, 2018; Marchi and Plastino, 2020; Sano et al., 2020). In brown algae, researchers have found mutants with impaired life cycles, abnormal cell differentiation or with defects in cell elongation and higher growth rates (Peters et al., 2008; Coelho et al., 2011; Le Bail et al., 2011; Hirano et al., 2020; Sato et al., 2021). It is worth noting that for most of these mutants the causative mutations remain unknown, underscoring the need for reverse genetics tools in macroalgae research.

**TABLE 1:** Status of macroalgae research. This table provides a non-exhaustive overview of key macroalgae research domains discussed in this review, together with selected species. Superscript numbers (^1–45^) link to references detailed below table.

	Life cycle under control	Genetic resources: Mutant studies conducted genome sequenced		Transformation protocols available	Proof-of-concept for genome editing
**Phaeophyta** 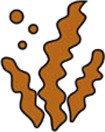	- *Alaria esculenta* ^30^	- *Ectocarpus* spp. 7^1^	- *Alaria esculenta* ^30^	- *Ectocarpus* spp. 7 ^(T),^ ^4^	- *Ectocarpus* spp. 7^4^
	- *Cladosiphon okamuranus* ^33^		- *Cladosiphon okamuranus* ^33^	- *Saccharina japonica* ^(T+S),^ ^25^^,^^26^	- *Saccharina japonica* ^3^
	- *Ectocarpus* spp. 7^1^		- *Ectocarpus* spp. 7^29^	- *Undaria pinnatifida* ^(S),^ ^5, 21^	
	- *Ectocarpus subulatus* ^31^		- *Ectocarpus subulatus* ^31^		
	- *Nemacystus decipiens* ^34^	- *Undaria pinnatifida* ^5^	- *Nemacystus decipiens* ^34^		
	- *Saccharina japonica* ^2, 3^		*- Saccharina japonica* ^35^		
	- *Sargassum fusiforme* ^36^		- *Sargassum fusiforme* ^36^		
	- *Undaria pinnatifida* ^5^		- *Undaria pinnatifida* ^32^		
**Chlorophyta** 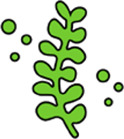	*- Caulerpa taxifolia* ^6^	*- Caulerpa taxifolia* ^6^	*- Caulerpa lentillifera* ^37^	- *Ulva lactuca* ^(T)^ ^7^	*- Ulva prolifera* ^14^
	- *Ulva compressa* ^38^	*- Ulva mutabilis* ^8^^,^^9^	*- Ulva compressa* ^38^	*- Ulva mutabilis* ^(S)^ ^10^	
	*- Ulva lactuca* ^7^	- *Ulva pertusa* ^11^	*- Ulva mutabilis* ^39^	- *Ulva pertusa* ^(T)^ ^12^	
	*- Ulva mutabilis* ^8^^,^^9^	*- Ulva rigida* ^15^	*- Ulva prolifera* ^40^		
	- *Ulva pertusa* ^11, 12^				
	*- Ulva prolifera* ^13^				
	*- Ulva rigida* ^15^				
**Rhodophyta** 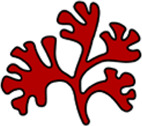	*- Chondrus cripus* ^16^	*- Chondrus cripus* ^16^	*- Chondrus cripus* ^41^	*- Kappaphycus alvarezii* ^(T),^ ^20^	
	*- Gracilaria birdae* ^17^	*- Gracilaria birdae* ^17^	- *Gracilaria changii* ^42^	*- Porphyra yezoensis* ^(T), 22^	
	*- Gracilaria caudata* ^18^	*- Gracilaria caudata* ^18^	- *Gracilariopsis chorda* ^43^	*- Pyropia yezoensis* ^(T+S), 27^^,^^28^	
	- *Gracilaria changii* ^42^	*- Gracilaria tikvahiae* ^19^	- *Porphyra umbilicalis* ^44^		
	- *Gracilariopsis chorda* ^43^	*- Porphyra yezoensis* ^22^	- *Pyropia yezoensis* ^45^		
	*- Gracilaria tikvahiae* ^19^	*- Pyropia kinositae* ^23^			
	*- Kappaphycus alvarezii* ^20^	*- Pyropia yezoensis* ^24^			
	- *Porphyra umbilicalis* ^44^				
	*- Porphyra yezoensis* ^22^				
	*- Pyropia kinositae* ^23^				
	- *Pyropia yezoensis* ^24^				

^T^ denotes transient transformation, ^S^ stable transformation, and ^T+S^ studies demonstrating both types. ^1^: Peters et al., 2008; ^2^: Song et al., 1998; ^3^: Shen et al., 2023; ^4^: Badis et al., 2021; ^5^: Sato et al., 2021; ^6^: Jongma et al., 2013; ^7^: Huang et al., 1996; ^8^: Bryhni, 1974; ^9^: Løvlie, 1969; ^10^: Oertel et al., 2015; ^11^: Kakinuma et al., 2006; ^12^: Kakinuma et al., 2009; ^13^: He et al., 2021; ^14^: Ichihara et al., 2021; ^15^: Gao et al., 2017; ^16^: Cornish et al., 2013; ^17^: Plastino et al., 2004; ^18^: Marchi and Plastino 2020; ^19^: Kursar et al., 1983; ^20^: Wang et al., 2010; ^21^: Song et al., 2003; ^22^: Mei et al., 1998; ^23^: Sano et al., 2020; ^24^: Lee and Choi, 2018; ^25^: Jiang et al., 2003; ^26^: Zhang et al., 2008; ^27^: Hirata et al., 2014; ^28^: Uji et al., 2014; ^29^: Cock et al., 2010; ^30^: Bringloe et al., 2021; ^31^: Dittami et al., 2020; ^32^: Graf et al., 2021; ^33^: Nishitsuji et al., 2016; ^34^: Nishitsuji et al., 2019; ^35^: Liu et al., 2019; ^36^: Wang et al., 2020; ^37^: Arimoto et al., 2019; ^38^: Osorio et al., 2022; ^39^: de Clerck et al., 2018; ^40^: He et al. 2021; ^41^: Collén et al., 2013; ^42^: Ho et al., 2018; ^43^: Lee et al., 2018; ^44^: Brawley et al., 2017; ^45^: Nakamura et al., 2013; Bringloe et al., 2021 (Nishitsuji et al., 2016; Nishitsuji et al., 2019; Dittami et al., 2020) (T. Liu et al., 2019; Wang et al., 2020; Osorio et al., 2022; He et al., 2021; Collén et al., 2013; Ho et al., 2018) (J. Lee et al., 2018; Brawley et al., 2017).

A major bottleneck for macroalgal genetic research is the lack of protocols for generating transgenic organisms (Table 1). Already in 1998, Song and co-workers reported the stable transformation of the brown seaweed *Laminaria japonica*, but few reports have been published after that. It is only in recent years that there has been a renewed interest in optimizing transformation protocols. One recently published cloning toolkit was designed to stably express nuclear genes in the green seaweed *Ulva mutabilis/compressa* (Blomme et al., 2021). This toolkit allows to overexpress transgenes and tagged endogenous genes. In the red seaweed *P. yezoensis*, overexpression of endogenous genes has proven to be feasible by Zheng et al. (2022). Another report by Cao et al. (2022), optimized a biolistic protocol to allow a high-efficiency stable transformation in this species. However, it should be noted that only reporter genes were used in this study. Despite these successes, only a transient expression of transgenes is typically achieved in macroalgae (reviewed in Mikami (2013)). Consequently, there is a paucity of reports describing successful stable transformation in macroalgae. Hurdles in stable transgene expression in macroalgae include the identification of a selection system, overcoming transgene silencing and the identification of regulatory sequences (Stock et al., 2024).

## 2 Genome editing in macroalgae: The current state

At least on paper, seaweeds are attractive organisms for genome engineering. Typically, they exhibit advantageous characteristics such as a multicellular haploid life stage, relatively small genomes with few duplication events, and the production of prodigious amounts of (a)sexual spores/gametes (Stock et al., 2024). Although not all life cycles in this very diverse group of organisms are easy to complete in the lab (Table 1), some life cycles are also relatively short. *Ulva mutabilis*, for example, has a cycle that can be completed in less than 2 months, putting it on par with the plant model *Arabidopsis thaliana* (Krämer, 2015). Despite these favorable traits, successful reports of genome editing in macroalgae are very scarce, much like stable transformation protocols.

Homologous recombination is a commonly used system for gene editing in animals, bacteria, and fungi (Aguilera and Carreira, 2021). However, there are no reports of this approach being functional in macroalgae, with the sole exception being a plastid gene expression system in *Pyropia yezoensis*. This system, however, does not permit alterations of the nuclear genome (Kong et al., 2017). More recent additions to the bioengineer’s toolbox such as Meganucleases, ZFNs (Zinc Finger Nucleases), TALENs (Transcription Activator-Like Effector Nucleases) and CRISPR (Clustered Regularly Interspaced Short Palindromic Repeats) systems have been applied in about 10 microalgae genera (Sizova et al., 2013; Daboussi et al., 2014; Greiner et al., 2017; Jeong et al., 2023), but only CRISPR has been tested in macroalgae.

Three reports have demonstrated the successful application CRISPR technology in macroalgae: the brown algae *Ectocarpus* species 7 (Badis et al., 2021) and *Saccharina japonica* (Shen et al., 2023), as well as in the green seaweed *Ulva prolifera* (Ichihara et al., 2022) (Table 1). All these studies made use of CRISPR-Cas9 ribonucleoproteins (RNPs), which consist of preassembled Cas9 protein-gRNA complexes. This demonstrates that a functional stable transformation system is not a prerequisite to successfully make use of CRISPR in seaweeds. Indeed, this DNA-free method may even offer advantages in terms of efficiency, as well as in compliance with legislative and regulatory requirements (Woo et al., 2015). Additionally, there is no need to optimize transgene expression which is often a bottleneck in macroalgae (Blomme et al., 2021). *ADENINE PHOSPHORIBOSYL TRANSFERASE* (*APT*) was selected as the primary target in all three studies. This conserved gene is involved in the salvage pathway of adenine, and the enzyme encoded by this gene uses adenine or analogues thereof as its substrate. When the analogue 2-fluoroadenine (2-FA) is applied, it is converted into toxic nucleotides in wild type cells, but not in cells containing a knockout of this gene (Schaff, 1994). The *Ectocarpus* species 7 study (Badis et al., 2021) went one step further, and utilized a co-targeting approach to enrich for modifications at a second locus (Kim et al., 2014; Mikkelsen and Bak, 2023). This approach led to the isolation of double mutants for three different secondary loci at frequencies between 0% and 100% of the 2-FA resistant population. This stark difference in editing outcomes may be accounted for by differences in gRNA efficiency, which are generally not easy to predict *in silico* (Konstantakos et al., 2022). Nevertheless, this study clearly shows the potential of using *APT* as a selectable marker to enrich for mutants in a target of interest. With biolistically transformed gametes, the editing efficiency was approximately 2 × 10^−5^ for single mutants and 2.5 × 10^−6^ for double mutants under the most favorable experimental conditions reported. When microinjecting unilocular sporangia—which typically develop a minimum of 100 haploid spores—higher transformation efficiencies of approximately 4%–7% for single mutants and 0.3% for double mutants were observed. Despite these rather low efficiencies, double mutants could be generated in 80% of the experiments. In *S. japonica* (Shen et al., 2023), the reported *APT* editing efficiencies were remarkably high, 8.6% and 4.5% for the microinjected female and male gametophytes, respectively. The higher efficiency in female gametophytes was attributed to their larger cell size which potentially minimizes injection damage. The *U. prolifera* study (Ichihara et al., 2022) made use of polyethylene glycol (PEG)-mediated transfection of gametes and reported *APT* mutation efficiencies between 1.6 × 10^−1^ and 3.0 × 10^−3^, and due to the massive number of initial gametes (1.0 × 10^6^), successful CRISPR events could be detected in all experiments. All three studies reported small indel mutations (<10 bp) as the primary editing outcome. Additionally, in the *S. japonica* study (Shen et al., 2023) two instances of larger deletions were observed among 35 mutants, one being 35 base pairs and the other 60 base pairs in length. Notably, the *U. prolifera* study (Ichihara et al., 2022) also identified substitution mutations. For one specific gRNA, substitutions–with no accompanying indels–were observed in as many as 16% of the mutants (2 out of 12 individuals).

Taken together, these publications demonstrate the successful application of genome editing in both brown and green seaweed species. It is important to note, however, that these reports were limited to proof-of-concept studies. Further research is needed to increase the mutation efficiencies and broaden the applicability of targeted mutagenesis systems in seaweeds.

## 3 Charting the way forward

**FIGURE 1:**
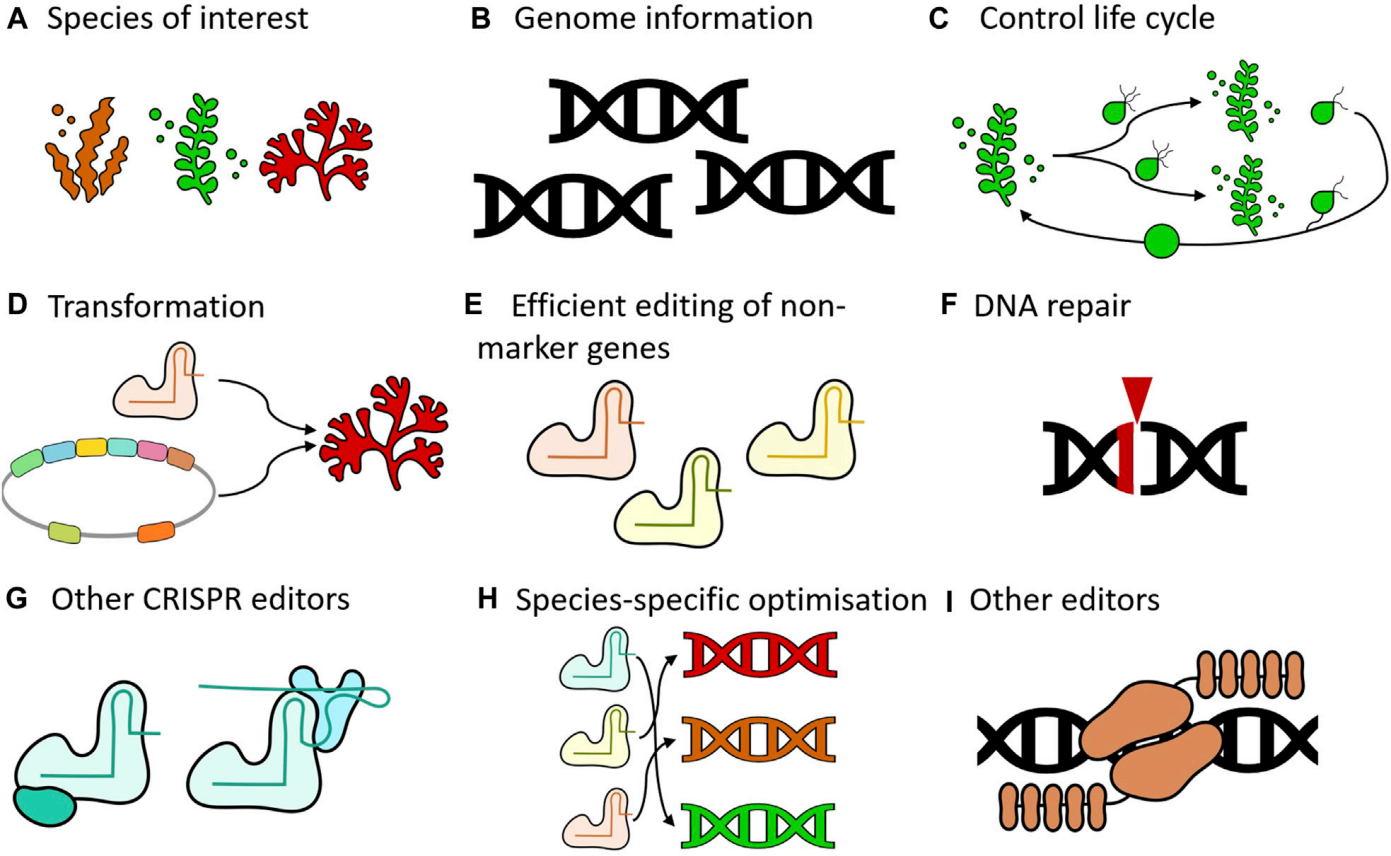
Key areas for advancing seaweed genome editing. **(A)** Species of interest. Expansion of the range of species that are amenable to genome editing would be desirable. Genome editing has not been demonstrated in any red seaweed, for example. **(B)** Genome information. Having access to genomic information of target species is critical for implementing genome editing technology as this will not only inform on the design of the reagents but will also contribute to avoiding off-target effects. **(C)** Control life cycle. Completing the full life cycle under laboratory conditions is desirable to advance supporting technologies (e.g., genetic transformation) and genome editing. **(D)** Transformation. Various methods can be utilized to introduce reagents into organisms, including electroporation, microinjection, transfection, and *Agrobacterium*-mediated transformation. The establishment of functional systems, whether transient or stable, is essential for genome editing. **(E)** Efficient editing of non-marker genes. All published studies targeted *ADENINE PHOSPHORIBOSYL TRANSFERASE* (*APT*) as the only gene or to enrich mutants at a second locus. Future efforts should focus on developing methods to generate mutants without the need for such enrichment processes. **(F)** DNA repair. Nucleases create double-stranded breaks in the DNA, which subsequently activate repair mechanisms. Gaining deeper insights into the specific repair processes active in the target species will guide the selection of appropriate genome editing strategies. **(G)** Other CRISPR editors. CRISPR mediated knock-outs are a very powerful tool, but other modalities such as base editing and prime editing can also be explored. Depending on the repair mechanisms that are active, these systems may be more efficient than conventional CRISPR systems **(H)** Species-specific optimization. Genome editing tools do not operate with the same efficiency across different organisms. Species-specific optimization can be beneficial to address this variability. **(I)** Other editors can also be used. This includes systems that already exist (e.g., TALENs), as well as systems that are yet to be developed.

Indeed, there are still some shortcomings that need to be addressed to make genome editing a viable technology in seaweeds. As previously noted, genome editing has been successfully applied in only a select few species (Table 1). To broaden the applicability of this technology (Figure 1A), more genomes of seaweed species will need to become available (Figure 1B). Ideally, these species should have life cycles that can be completed in laboratory settings (Figure 1C), allowing genetic transformation protocols to be developed (Figure 1D). Another limitation is that in all studies published until now, *APT* was either the sole gene that was targeted or was used to enrich mutants at a second locus (Figure 1E). While a knockout in *APT* is not lethal, it is likely that there will be an effect on the fitness of these organisms (Liu et al., 2023). Genetic interactions with other genes can also not be excluded, which may be an issue for functional characterization of other loci. A major challenge is that the reported mutation efficiencies are very low, except for *S. japonica*. Even in this species, the creation of double or higher order mutants may be difficult to achieve using the published protocol. In general, some kind of selection will remain necessary in absence of a drastic increase in editing efficiencies.

To improve the efficiency of genome editing in seaweed, several approaches may be utilized. We will focus here on CRISPR systems, as these systems are currently unparalleled in knocking out genes in a variety of organisms. A first important consideration is that these nucleases will create double-stranded breaks, which will then need to be repaired by the cell. Therefore, the importance of the DNA repair mechanism is paramount (Figure 1F). This is a largely uncharted water, as these mechanisms have not been studied in detail in macroalgae. As previously discussed, genome editing results showed significant variation among the three seaweed species tested to date. Notably, *U. prolifera* exhibited a high incidence of substitution mutations, which is extremely rare in plants or animals (Hwang et al., 2020; Zhang et al., 2020). A lot of work on DNA repair has been done in other organisms, with many published protocols that can be leveraged here as well (Bjergbæk, 2016; Zentout et al., 2021; van de Kooij and van Attikum, 2022). In mammalian systems the successful redirection of DNA repair pathways has already been demonstrated with chemical inhibitors (Maruyama et al., 2015), cell cycle synchronization (Lin et al., 2014), Homology-directed repair (HDR) template modifications (Cruz-Becerra and Kadonaga, 2020; Schubert et al., 2021), modulation of regulatory factors (Canny et al., 2018; Charpentier et al., 2018; Jayavaradhan et al., 2019), and engineered Cas9 variants (Chauhan et al., 2023). In this respect, having a functional DNA based - either stable or transient - transformation system would be a definite advantage. The ribonucleoproteins (RNPs) technology that was used in all studies to date depends on the availability of a recombinant Cas9 protein. While the standard purified Cas9 protein is commercially available, creating new variants or fusions would require the challenging and expensive task of cloning and purifying new proteins. Using a DNA based system would also allow for other CRISPR systems to be easily tested, such as base editors (Figure 1G). Gaillochet et al. (2023) for example, utilized a DNA-based high-throughput platform to optimize base editors for rapid implementation in plants. This would have been very difficult indeed if a protein-based platform was used instead. Base editors are an interesting technology, not only because they avoid the induction of double-stranded breaks, but also because they rely on other DNA repair mechanisms (Gu et al., 2021). In bacteria, for example, these chromosomal breaks are typically lethal to the cell, and here base editors offer a very efficient alternative to achieve genome editing (Cui and Bikard, 2016; Zheng et al., 2018; Rodrigues et al., 2021). Another variant in genome editing technology are prime editors. In this approach, a nickase Cas9 is fused to a reverse transcriptase, enabling the incorporation of specific changes into the DNA guided by a prime editing gRNA. Prime editors are generally less efficient than base editors in all systems tested to date. However, prime editors offer more flexibility with regards to the possible genomic changes and make use of yet other repair systems (Chen and Liu, 2023). Given the variability in editing efficiencies across different organisms, further experimentation will be necessary to identify suitable systems for different groups of macroalgae (Figure 1H).

Here we focused on CRISPR based genome editing, but that does not mean that other technologies should be dismissed. TALENs, for example, have advantages in specificity and IP landscape compared to CRISPR (Cloney, 2016; Bhardwaj and Nain, 2021). As the genome editing field is developing at a rapid pace, new tools are also continuously being developed, which could offer benefits in terms of efficiency, adaptability, and precision (Figure 1I).

## 4 Navigating the applications of gene edited seaweeds

Seaweeds are economically important organisms. Processed red seaweed species in the genus *Pyropia* have a market value of about 2 billion USD in 2017 (San et al., 2023). Cultivars have been developed for economically important species such as *S. japonica*, *Kappaphycus alvarezii*, *Ulva* ssp., and *Gracilaria* spp. (Levy and Friedlander, 1990; Hayashi et al., 2007; Su et al., 2020; Lawton et al., 2021). Cultivars have been generated using a variety of techniques, including traditional selection, heavy ion radiation, and ethyl methanesulfonate (EMS) mutagenesis (Niwa et al., 2011; Park and Hwang, 2014; Lee and Choi, 2018; Hwang and Park, 2020; Kong et al., 2023). Nevertheless, whereas land plants which have been cultivated and gradually domesticated for more than 12,000 years (Purugganan, 2019), the earliest record of deliberate seaweed cultivation dates back to about 400 years ago in Korea (Hwang and Park, 2020). Modern seaweed cultivation has only started in the 1940s and currently still relies on relatively few species and cultivars (47 certified cultivars in 2019; Hwang et al., 2019). Breeding campaigns take a significant amount of time, partly because organisms obtained by mutation breeding methods are often burdened by background mutations that need to be removed by extensive backcrossing (Holme et al., 2019). Taken together, a substantial genetic potential is still untapped in a diverse group of organisms with clear commercial value.

Today, the increase in seaweed cultivation comes with a higher prevalence of poorly understood diseases and biofouling epiphytes, combined with abiotic challenges such as ocean acidification and increase in water temperature (Sugumaran et al., 2022). All these stressors impact yield negatively. As seaweed production can contribute to the United Nations sustainable development goals, establishing techniques like genome editing will be crucial to generate, *e.g.*, disease-resistant cultivars (Valero et al., 2017; Hayashi et al., 2020; Sugumaran et al., 2022). Therefore, exploiting genome editing systems may offer a fast way to produce elite strains with desired characteristics. Developing *de novo* domesticated plants through genome editing is not a pipedream and has been successfully demonstrated multiple times already. In these instances, closely related domesticated species, such as tomato (*Solanum lycopersicum*) and rice (*Oryza sativa*), harbored known domestication-related genes (Yu and Li, 2022). Although these types of genetic resources are not as well developed in seaweed species, Genome-Wide Association Studies (GWAS) have the potential to identify interesting candidate genes. These investigations have already yielded genetic regions associated with various yield-related traits in red and brown seaweeds (Liu et al., 2010; Xu et al., 2012; Shan et al., 2015; Avia et al., 2017; Huang and Yan, 2019; Hwang et al., 2019). Traits of interest for cultivar improvement can be disease resistance and yield, but can include nutrient content, production of metabolites or macromolecules, and tolerance to environmental conditions.

## 5 Discussion

The advent of CRISPR technology has revolutionized the life sciences (van der Oost and Patinios, 2023). Nevertheless, not all fields have been able to reap the promises that this genome editing tool holds. One such field is phycology, which has seen only three published reports on seaweed genome editing to date, despite the ecological and economic importance of these organisms. One major bottleneck is the absence of robust transformation protocols for many seaweed species, preventing the screening of gene editing reagents as is commonly done in contemporary experimental setups. This underscores the need for the development of genetic tools specifically tailored to these organisms. In the Environmental Model Systems (EMS) Project (Faktorová et al., 2020), researchers attempted to optimize transformation protocols in 39 marine protist species, which included green microalgae. The results showed that after optimization exogenous DNA could be successfully delivered and expressed in over 50% of these species. Importantly, no single universally applicable protocol was identified for all species. It should be noted however, that this study was conducted by no less than 113 authors, and a similar collaborative effort will be needed to advance the macroalgae field. Smaller-scale efforts can still benefit from considerable progress made in unicellular model systems such as *Chlamydomonas reinhardtii*, *Cyanidioschyzon merolae* or *Phaedactylum tricornutum*. For example, both *Ulva* and *Chlamydomonas* transformant selection relies on the same bleomycin resistance gene (Oertel et al., 2015) and several transit peptides isolated from *Chlamydomonas* are functional in *Ulva* (Blomme et al., 2021).

Successful development of genome editing techniques in macroalgae will not only yield insights into the biology of these organisms themselves but will also provide valuable information for understanding the biology of other groups, particularly land plants. This includes insights into the evolution of multicellularity (Coates et al., 2015; De Clerck et al., 2018), organogenesis (Bogaert et al., 2013; 2023), and phytohormone pathways (Bogaert et al., 2022). Additionally, genome editing will also enable the generation of customized strains which can be used in aquaculture. Given the current challenges of higher-intensity cultivation coupled with global change and concomitant effects such as ocean acidification (Sugumaran et al., 2022), breeding new varieties will be an important strategy to future-proof this industry.

In summary, while genome editing in macroalgae is still in its early stages, its potential impact is significant. Future efforts in the field should focus on improving not only genome editing protocols, but also other supporting biotechnological techniques.
